# Supplementary material for: Prodromal symptoms of rheumatoid arthritis in a primary care database: variation by ethnicity and socioeconomic status
Source: Rheumatology (Oxford). 2024 Mar 11;64(3):1029–35. doi: 10.1093/rheumatology/keae157 (PMC11879358; doi:10.1093/rheumatology/keae157)
Supplement: keae157_Supplementary_Data [file keae157_supplementary_data.zip › keae157_Supplementary_Data/rhe-23-2457-File002.docx]

Supplementary Data S1: Exploratory review of prodromal symptoms

An explorative literature search was conducted in August 2022 to create the baseline list of included prodromal symptoms of RA. The search included peer-reviewed, published scholarship. The below table outlines sources. Detailed references on next page.

| **Category** | **Variable** |
| --- | --- |
| Demographics | Sex (Crowson et al, 2011) |
|  | Age (Xu et al, 2021) |
|  | Family History of RA* (Kronzer et al, 2021) |
|  | BMI (Mankia et al, 2021) |
|  | Level of physical activity* (Sun et al, 2021) |
|  | Educational level (Socioeconomic status) (Xu et al, 2021) |
|  | Ethnicity (Xu et al, 2021) |
| Symptoms / signs | Joint problems (including pain and swelling): (Muller et al, 2019)   - Shoulder problems - Neck problems - Foot problems - Hand problems - Jaw problems |
|  | Musculoskeletal pathologies: (Muller et al, 2019)   - Frozen shoulder - Carpal Tunnel Syndrome - Palindromic Rheumatism |
|  | Morning stiffness (Muller et al, 2019) |
|  | Unintentional weight loss (Muller et al, 2019) |
|  | Difficulty making a fist, grip weakness* (Wouters et al, 2019) |
|  | Fatigue (Wouters et al, 2019) |
|  | Altered sensations (e.g. tingling , numbness, neuropathy)* (Stack et al, 2014) |
|  | Falls (Stack et al, 2014) |
|  | Weakness* (Stack et al, 2014) |

*Symptoms/demographics not recorded reliably in CRPD Aurum and therefore excluded from the study.

Crowson, C.S., Matteson, E.L., Myasoedova, E., Michet, C.J., Ernste, F.C., Warrington, K.J., Davis, J.M., III, Hunder, G.G., Therneau, T.M. and Gabriel, S.E. (2011), The lifetime risk of adult-onset rheumatoid arthritis and other inflammatory autoimmune rheumatic diseases. Arthritis & Rheumatism, 63: 633-639. <https://doi.org/10.1002/art.30155>

Khidir S, Wouters F, van der Helm-van Mil A, van Mulligen E. 2022. The course of fatigue during the development of Rheumatoid Arthritis and its relation with inflammation: a longitudinal study.Joint Bone Spine. https://doi.org/10.1016/j.jbspin.2022.105432.

Kronzer V, Crowson CS , Sparks JA , Myasoedova E, Davis J. (2021), Family History of Rheumatic, Autoimmune, and Nonautoimmune Diseases and Risk of Rheumatoid Arthritis. Arthritis Care Res, 73: 180-187. <https://doi.org/10.1002/acr.24115>

Lingling Sun, Jiahao Zhu, Yuxiao Ling, Shuai Mi, Yasong Li, Tianle Wang, Yingjun Li, Physical activity and the risk of rheumatoid arthritis: evidence from meta-analysis and Mendelian randomization, *International Journal of Epidemiology*, Volume 50, Issue 5, October 2021, Pages 1593–1603, <https://doi.org/10.1093/ije/dyab052>

Mankia K, Siddle H, Di Matteo A, et al. A core set of risk factors in individuals at risk of rheumatoid arthritis: a systematic literature review informing the EULAR points to consider for conducting clinical trials and observational studies in individuals at risk of rheumatoid arthritis. RMD Open 2021;7:e001768. doi: 10.1136/rmdopen-2021-001768

Muller, S., S. Hider, A. Machin, R. Stack, R. A. Hayward, K. Raza and C. Mallen (2019). "Searching for a prodrome for rheumatoid arthritis in the primary care record: A case-control study in the clinical practice research datalink." Semin Arthritis Rheum **48**(5): 815-820.

van de Stadt, L. A., B. I. Witte, W. H. Bos and D. van Schaardenburg (2013). "A prediction rule for the development of arthritis in seropositive arthralgia patients." Annals of the Rheumatic Diseases **72**(12): 1920-1926.

Nikolet K den Hollander, Marloes Verstappen, Navkiran Sidhu, Elise van Mulligen, Monique Reijnierse, Annette H M van der Helm-van Mil, Hand and foot MRI in contemporary undifferentiated arthritis: in which patients is MRI valuable to detect rheumatoid arthritis early? A large prospective study, Rheumatology, 2022;, keac017, https://doi.org/10.1093/rheumatology/keac017

Stack RJ, van Tuyl LH, Sloots M, van de Stadt LA, Hoogland W, Maat B, Mallen CD, Tiwana R, Raza K, van Schaardenburg D. Symptom complexes in patients with seropositive arthralgia and in patients newly diagnosed with rheumatoid arthritis: a qualitative exploration of symptom development. Rheumatology. 2014 Sep 1;53(9):1646-53.

Wouters F, van der Giesen FJ, Matthijssen XME*, et al* Difficulties making a fist in clinically suspect arthralgia: an easy applicable phenomenon predictive for RA that is related to flexor tenosynovitis. *Annals of the Rheumatic Diseases*2019;**78:**1438-1439.

Xia Feng, Xizhu Xu, Yanjun Shi, Xuezhen Liu, Huamin Liu, Haifeng Hou, Long Ji, Yuejin Li, Wei Wang, Youxin Wang, Dong Li, "Body Mass Index and the Risk of Rheumatoid Arthritis: An Updated Dose-Response Meta-Analysis", BioMed Research International, vol. 2019, Article ID 3579081, 12 pages, 2019. <https://doi.org/10.1155/2019/3579081>

Xu Y, Wu Q. Prevalence Trend and Disparities in Rheumatoid Arthritis among US Adults, 2005-2018. J Clin Med. 2021 Jul 26;10(15):3289. doi: 10.3390/jcm10153289. PMID: 34362073; PMCID: PMC8348893.

Supplementary Data S2: Included symptoms

Table of the 36 included prodromal RA symptoms, as defined by the preceding review by Muller et al (2019) (19) and modified as part of the exploratory review (see Supplementary material 1).

| **Localised musculoskeletal symptoms** | |
| --- | --- |
| Ankle issues | Jaw issues |
| *Ankle pain* | *Jaw pain* |
| *Ankle stiffness* | *Jaw stiffness* |
| *Ankle swelling* | Knee issues |
| Elbow issues | *Knee pain* |
| *Elbow pain* | *Knee stiffness* |
| *Elbow stiffness* | *Knee swelling* |
| *Elbow swelling* | Neck issues |
| Foot issues | *Neck pain* |
| *Foot pain* | *Neck stiffness* |
| *Foot stiffness* | Wrist issues |
| Hand and finger issues | *Wrist pain* |
| *Hand and finger pain* | *Wrist swelling* |
| *Hand and finger stiffness* | *Wrist stiffness* |
| *Hand and finger swelling* | Shoulder issues |
| Hip issues | *Shoulder pain* |
| *Hip pain* | *Shoulder stiffness* |
| *Hip stiffness* | *Shoulder swelling* |
| **Other musculoskeletal symptoms** | **Non-musculoskeletal symptoms** |
| *Joint swelling* | *Unintended weight loss* |
| *Unspecified muscle cramps* | *Fatigue* |
| *Morning stiffness* | *Night sweats* |
|  | *Gingivitis or periodontitis* |
|  | *Falls* |
|  | *Stress, unspecified* |
|  | *Rheumatic nodules* |

Supplementary Data S3: Example of code list

Example of code list to extract data from CPRD Aurum, below for symptom “Ankle Pain”. The full set of code lists is available on request from the corresponding author.

| **MEDICAL_CODE_ID** | **DESCRIPTION** | **READ_CODE** | **SNOMED_CT_CODE** | **FREQUENCY** | **DATABASE** |
| --- | --- | --- | --- | --- | --- |
| 310840017 | Ankle joint pain | N094711 | 202490009 | 65032 | CPRD_AURUM |
| 890671000006110 | Ankle/foot joint pain | N094799 | 267954009 | 16528 | CPRD_AURUM |
| 483791000006115 | Ankle pain | N245.11 | 247373008 | 1285109 | CPRD_AURUM |
| 369388017 | Ankle pain | 1M13.00 | 247373008 | 198771 | CPRD_AURUM |
| 11902321000006100 | Subtalar joint pain |  | 202491008 | 253 | CPRD_AURUM |
| 5502581000006110 | Ankle and/or foot joint pain |  | 267954009 | 20 | CPRD_AURUM |
| 310842013 | Arthralgia of subtalar joint | N094Q00 | 202491008 | 882 | CPRD_AURUM |
| 12224031000006100 | Tenderness of ankle joint |  | 299446004 | 3 | CPRD_AURUM |
| 12224071000006100 | Tenderness of subtalar joint |  | 299553005 | 1 | CPRD_AURUM |
| 8018171000006110 | Chronic ankle pain |  | 51881000119109 | 5 | CPRD_AURUM |
| 5867071000006110 | Ankle joint - painful on movement |  | 299447008 | 38 | CPRD_AURUM |

Supplementary Data S4: Supplementary analyses

a) Odds ratio of any symptom in study population and matched controls

The study population is compared with a matched control group extracted from the same CRPD Aurum dataset, matched by age (±1 year), sex and primary care practice but without a diagnosis of RA. The analyses were adjusted logistic regressions with “having any of the included 21 symptoms” as the binary dependent, ethnicity as independent, and age, sex, BMI and smoking status as covariates in the same manner as the main analysis. This was conducted separately for individuals with RA and matched controls without RA. Note that socioeconomic status/IMD is not included, due to these data not being available for the control group at the time of analysis. This explains why the sample size in the exposed population is higher than the sample in the main analysis (no cases excluded due to lack of IMD data). However, the main analysis indicated that IMD had very limited impact on the OR of reporting symptoms of prodromal RA.

Coded symptoms were overrepresented in cases of Black and South Asian ethnicity to a similar degree in the control population as in the exposed cohort. However, the overall symptom prevalence was much lower at 24.1 % (all ethnic groups) in the control group compared to the RA study population at 48.9 %, suggesting that the differences in symptoms found in this study can be attributed to not only differences in baseline symptoms, but actual differences in prodromal RA symptoms.

Table: Adjusted odds ratio of having any of the 21 included symptoms in the study population and in a non-RA control group matched by age (+- 1 year), sex and primary care practice.

|  | **Ethnicity** | **OR** | **p** | **95 % CI** | |
| --- | --- | --- | --- | --- | --- |
| **Exposed** | *Black* | 1.21* | <0.01 | 1.10 | 1.32 |
| n=71320  Prevalence of any symptom:  48.9 % (n=34687) | *Mixed* | 0.95 | 0.53 | 0.81 | 1.11 |
|  | *Others* | 1.10 | 0.29 | 0.92 | 1.32 |
|  | *South Asian* | 1.18* | <0.01 | 1.11 | 1.25 |
|  | *White* | 1.00 | (base) |  |  |
|  |  |  |  |  |  |
| **Controls** | *Black* | 1.12* | 0.03 | 1.01 | 1.24 |
| n=67483  Prevalence of any symptom:  24.1 % (n=16257) | *Mixed* | 1.06 | 0.58 | 0.87 | 1.28 |
|  | *Others* | 0.98 | 0.87 | 0.79 | 1.22 |
|  | *South Asian* | 1.35* | <0.01 | 1.26 | 1.45 |
|  | *White* | 1.00 | (base) |  |  |

OR = Odds Ratio. CI = Confidence Interval. RA = Rheumatoid Arthritis.
* Statistically significant at p<0.05.

b) Comparison of adjusted and non-adjusted regression model

Results of regression model for odds ratio (OR) of having any of the 21 included symptoms. Adjusted model includes BMI, sex, age and smoking status. Asterix denotes statistical significance (p<0.05). The exclusion of confounders (non-adjusted analysis) did not affect the results to a significant degree.

| **Adjusted** | **OR** | **p** | **95 % CI** |  |
| --- | --- | --- | --- | --- |
| Black | 1.17 | 0.00 | 1.07 | 1.29 |
| Mixed | 1.06 | 0.55 | 0.88 | 1.28 |
| Others | 0.99 | 0.91 | 0.84 | 1.17 |
| South Asian | 1.16 | 0.00 | 1.09 | 1.23 |
| White | 1.00 | . | . | . |
| IMD q5 | 1.00 | . | . | . |
| IMD q4 | 1.03 | 0.27 | 0.98 | 1.08 |
| IMD q3 | 1.01 | 0.59 | 0.97 | 1.06 |
| IMD q2 | 0.99 | 0.63 | 0.94 | 1.04 |
| IMD q1 | 1.01 | 0.58 | 0.97 | 1.06 |
| **Unadjusted** | **OR** | **p** | **95 % CI** |  |
| Black | 1.17 | 0.00 | 1.07 | 1.28 |
| Mixed | 1.02 | 0.85 | 0.85 | 1.23 |
| Others | 0.96 | 0.65 | 0.82 | 1.13 |
| South Asian | 1.11 | 0.00 | 1.05 | 1.18 |
| White | 1.00 | . | . | . |
| IMD q5 | 1.00 | . | . | . |
| IMD q4 | 1.03 | 0.27 | 0.98 | 1.08 |
| IMD q3 | 1.02 | 0.47 | 0.97 | 1.07 |
| IMD q2 | 0.99 | 0.80 | 0.95 | 1.04 |
| IMD q1 | 1.03 | 0.25 | 0.98 | 1.08 |

OR = Odds Ratio. CI = Confidence Interval. IMDq = IMD quintile.

c) Stratified regression by ethnicity and by IMD quintile

Regression model for presence of any of the 21 included symptoms, exploring association with IMD quintile stratified by ethnicity (left) and association with ethnicity stratified by IMD quintile (right). Significance set to p<0.05. The stratification by ethnicity and IMD indicated that IMD has little impact on coded symptoms and that the larger effect lies with ethnicity.

| **Stratified by ethnicity, adjusted.** | | | | |  | **Stratified by IMD quintile (IMD q), adjusted.** | | | | |
| --- | --- | --- | --- | --- | --- | --- | --- | --- | --- | --- |
| **BLACK** | or | P | 95% CI | |  | **IMD q5 (least deprived)** | OR | p | 95% CI | |
| IMD q5 | 1 |  |  |  |  | Black | 1.614 | 0.048 | 0.864 | 3.015 |
| IMD q4 | 1.029 | 0.081 | 0.268 | 1.288 |  | Mixed | 0.715 | 0.308 | 0.306 | 1.669 |
| IMD q3 | 1.022 | 0.146 | 0.337 | 1.353 |  | Others | 1.24 | 0.388 | 0.653 | 2.355 |
| IMD q2 | 1.004 | 0.381 | 0.415 | 1.542 |  | South Asian | 1.212 | 0.03 | 0.965 | 1.521 |
| IMD q1 | 1.031 | 0.293 | 0.398 | 1.473 |  | White | 1 | . | . | . |
|  |  |  |  |  |  |  |  |  |  |  |
| **SOUTH ASIAN** |  |  |  |  |  | **IMD q4** |  |  |  |  |
| IMD q5 | 1 |  |  |  |  | Black | 0.91 | 0.608 | 0.567 | 1.461 |
| IMD q4 | 1.198 | 0.672 | 0.399 | 3.597 |  | Mixed | 0.83 | 0.474 | 0.426 | 1.619 |
| IMD q3 | 1.203 | 0.643 | 0.431 | 3.356 |  | Others | 0.978 | 0.911 | 0.589 | 1.625 |
| IMD q2 | 1.662 | 0.183 | 0.622 | 4.442 |  | South Asian | 1.262 | 0.004 | 1.024 | 1.555 |
| IMD q1 | 1.972 | 0.082 | 0.721 | 5.388 |  | White | 1 | . | . | . |
|  |  |  |  |  |  |  |  |  |  |  |
| **WHITE** |  |  |  |  |  | **IMD q3** |  |  |  |  |
| IMD q5 | 1 |  |  |  |  | Black | 1.027 | 0.824 | 0.757 | 1.393 |
| IMD q4 | 1.028 | 0.257 | 0.965 | 1.096 |  | Mixed | 0.9 | 0.604 | 0.533 | 1.52 |
| IMD q3 | 1.018 | 0.477 | 0.954 | 1.088 |  | Others | 0.973 | 0.885 | 0.603 | 1.572 |
| IMD q2 | 0.976 | 0.357 | 0.914 | 1.044 |  | South Asian | 1.142 | 0.049 | 0.96 | 1.358 |
| IMD q1 | 1.023 | 0.386 | 0.957 | 1.093 |  | White | 1 | . | . | . |
|  |  |  |  |  |  |  |  |  |  |  |
| **MIXED** |  |  |  |  |  | **IMD q2** |  |  |  |  |
| IMD q5 | 1 |  |  |  |  | Black | 0.91 | 0.608 | 0.567 | 1.461 |
| IMD q4 | 1.198 | 0.672 | 0.399 | 3.597 |  | Mixed | 0.83 | 0.474 | 0.426 | 1.619 |
| IMD q3 | 1.203 | 0.643 | 0.431 | 3.356 |  | Others | 0.978 | 0.911 | 0.589 | 1.625 |
| IMD q2 | 1.662 | 0.183 | 0.622 | 4.442 |  | South Asian | 1.262 | 0.004 | 1.024 | 1.555 |
| IMD q1 | 1.972 | 0.082 | 0.721 | 5.388 |  | White | 1 | . | . | . |
|  |  |  |  |  |  |  |  |  |  |  |
| **OTHER** |  |  |  |  |  | **IMD q1 (most deprived** |  |  |  |  |
| IMD q5 | 1 |  |  |  |  | Black | 1.614 | 0.391 | 0.864 | 3.015 |
| IMD q4 | 0.835 | 0.576 | 0.364 | 1.917 |  | Mixed | 0.715 | 0.235 | 0.306 | 1.669 |
| IMD q3 | 0.817 | 0.519 | 0.364 | 1.832 |  | Others | 1.24 | 0.309 | 0.653 | 2.355 |
| IMD q2 | 0.839 | 0.556 | 0.388 | 1.811 |  | South Asian | 1.212 | 0.107 | 0.965 | 1.521 |
| IMD q1 | 0.802 | 0.474 | 0.362 | 1.775 |  | White | 1 | . | . | . |

IMD q = Indices of Multiple Deprivation, quintile. CI = Confidence Interval.
